# Multifunctionality of Reduced Graphene Oxide in Bioderived Polylactide/Poly(Dodecylene Furanoate) Nanocomposite Films

**DOI:** 10.3390/molecules26102938

**Published:** 2021-05-15

**Authors:** Giulia Fredi, Mahdi Karimi Jafari, Andrea Dorigato, Dimitrios N. Bikiaris, Riccardo Checchetto, Matteo Favaro, Roberto Sennen Brusa, Alessandro Pegoretti

**Affiliations:** 1 Department of Industrial Engineering and INSTM Research Unit, University of Trento, Via Sommarive 9, 38123 Trento, Italy; mahdi.karimijafari@studenti.unitn.it (M.K.J.); andrea.dorigato@unitn.it (A.D.); matteo.favaro@unitn.it (M.F.); alessandro.pegoretti@unitn.it (A.P.); 2 Laboratory of Polymer Chemistry and Technology, Chemistry Department, Aristotle University of Thessaloniki, 54124 Thessaloniki, Greece; dbic@chem.auth.gr; 3 Department of Physics, University of Trento, Via Sommarive 14, 38123 Trento, Italy; riccardo.checchetto@unitn.it (R.C.); robertosennen.brusa@unitn.it (R.S.B.); 4 Trento Institute of Fundamental Physics and Applications, Via Sommarive 14, 38123 Trento, Italy

**Keywords:** poly(dodecylene furanoate) polylactic acid, biopolymers, reduced graphene oxide, nanocomposites, gas phase permeation

## Abstract

This work reports on the first attempt to prepare bioderived polymer films by blending polylactic acid (PLA) and poly(dodecylene furanoate) (PDoF). This blend, containing 10 wt% PDoF, was filled with reduced graphene oxide (rGO) in variable weight fractions (from 0.25 to 2 phr), and the resulting nanocomposites were characterized to assess their microstructural, thermal, mechanical, optical, electrical, and gas barrier properties. The PLA/PDoF blend resulted as immiscible, and the addition of rGO, which preferentially segregated in the PDoF phase, resulted in smaller (from 2.6 to 1.6 µm) and more irregularly shaped PDoF domains and in a higher PLA/PDoF interfacial interaction, which suggests the role of rGO as a blend compatibilizer. rGO also increased PLA crystallinity, and this phenomenon was more pronounced when PDoF was also present, thus evidencing a synergism between PDoF and rGO in accelerating the crystallization kinetics of PLA. Dynamic mechanical thermal analysis (DMTA) showed that the glass transition of PDoF, observed at approx. 5 °C, shifted to a higher temperature upon rGO addition. The addition of 10 wt% PDoF in PLA increased the strain at break from 5.3% to 13.0% (+145%), and the addition of 0.25 phr of rGO increased the tensile strength from 35.6 MPa to 40.2 MPa (+13%), without significantly modifying the strain at break. Moreover, rGO decreased the electrical resistivity of the films, and the relatively high percolation threshold (between 1 and 2 phr) was probably linked to the low aspect ratio of rGO nanosheets and their preferential distribution inside PDoF domains. PDoF and rGO also modified the optical transparency of PLA, resulting in a continuous decrease in transmittance in the visible/NIR range. Finally, rGO strongly modified the gas barrier properties, with a remarkable decrease in diffusivity and permeability to gases such as O_2_, N_2,_ and CO_2_. Overall, the presented results highlighted the positive and sometimes synergistic role of PDoF and rGO in tuning the thermomechanical and functional properties of PLA, with simultaneous enhancement of ductility, crystallization kinetics, and gas barrier performance, and these novel polymer nanocomposites could thus be promising for packaging applications.

## 1. Introduction

Bioplastics, defined as plastics that are derived from renewable resources and/or biodegradable, represent a promising alternative to traditional petroleum-derived polymers [1,2,3]. Not only can bioplastics show alternative disposal pathways, thus limiting the amount of plastic waste ending up in our environment, but they also allow a considerable reduction in carbon footprint throughout the whole life cycle, from resources extraction to the end of life [4]. These inherent advantages have raised academic and industrial interest towards bioplastics in recent decades, and a substantial effort is being made to translate their intrinsic benefits into applications that are not only more sustainable but also equally or more efficient than those involving traditional plastics. Biodegradable bioplastics are currently employed in many different fields and are especially interesting for single-use packaging, which accounts for the largest fraction in plastic waste [4]. Although bioplastics have been synthesized and investigated for nearly a century, their extensive industrialization is still at its infancy. Global bioplastics production in 2019 was 2.11 million tonnes (Mt), which represents a minimal fraction (i.e., 0.6%) of the global production of all plastics in the same year (359 Mt) [4]. As the world urgently needs a credible alternative to petroleum-based plastics, the market of bioderived and biodegradable plastics is expected to grow in the coming years [4]. However, the full exploitation of the potential of bioplastics is subordinate to the identification of suitable applications in order to optimize material properties with sustainable additives and fillers and address the main shortcomings that limit their applicability.

For example, one of the most interesting and investigated biopolymers is polylactic acid or polylactide (PLA), a thermoplastic biodegradable linear aliphatic polyester obtainable from renewable resources, such as corn and potato starch [5,6,7]. PLA exhibits high elastic modulus (2–3 GPa) and mechanical strength (40–60 MPa), good processability, and high optical transparency, and therefore, it is widely commercialized for packaging and textile applications [5,8,9]. However, the utilization of PLA in the packaging industry is generally limited to rigid thermoformed items, whereas the application of PLA as a flexible film is limited by its poor deformation at break and toughness, high sensitivity to moisture and relatively low gas barrier properties [6]. To tackle the intrinsic shortcomings of PLA, and more generally to tailor its physical and mechanical properties, one of the most efficient and inexpensive methods is polymer blending [10,11]. As reported in a recent review by Nofar et al. [12], PLA has been blended with several fossil-based and biobased polymers, such as poly(ethylene terephthalate) (PET) [13], poly(3-hydroxybutyrate) (PHB), and polycaprolactone (PCL) [14,15], with the aim of improving its ductility and gas barrier properties especially.

An interesting group of biopolymers that are blendable with PLA is that of poly(alkylene furanoate)s (PAFs). They are synthesized from the polycondensation between an alkylene glycol and 2,5-furandicarboxylic acid (FDCA), which was listed among the top 12 high-value-added chemicals from biorefinery of carbohydrates by the United States Department of Energy in 2004 [16] and 2010 [17]. PAFs represent the most promising bioderived alternative to fossil-based poly(alkylene terephthalates) (PATs), as they show thermomechanical and gas barrier properties similar or superior to those of the corresponding PATs, which makes them suitable for packaging applications [18,19,20,21,22]. The most extensively investigated PAF is poly(ethylene furanoate) (PEF), studied as the biobased alternative to poly(ethylene terephthalate) (PET) [23,24]. However, furan-based polyesters have also been synthesized with longer-chain diols, containing up to 12 carbon atoms [21]. An increase in the length of the diol alkyl chain promotes an enhancement of the molecular mobility, which leads to a decrease in the glass transition and melting temperatures and an increase in crystallization kinetics and ductility [21,25].

The published works on long-alkyl-chain (8 to 12 carbon atoms) PAFs are mostly focused on optimizing the synthesis route and investigating their thermal properties [21,26,27]. On the other hand, very few works aim to study their mechanical and gas barrier properties and to optimize their performance through blending and additivation. Our group has recently investigated the thermomechanical properties of nanocomposites based on poly(decylene furanoate) (PDeF) and carbon nanotubes (CNTs) [28]. More surprisingly, very few works deal with the preparation of PLA/PAF polymer blends, especially with long-alkyl-chain PAFs. Our group has recently prepared novel bioderived films through blending PLA with PAFs with varying alkyl chain lengths (4 to 10 carbon atoms). That work showed that the addition of a small (5–10 wt%) fraction of PAF into PLA promoted a remarkable increase in strain at break and fracture toughness. This phenomenon, occurring with long-alkyl-chain PAFs especially, led to polymer films being obtained with balanced properties, very promising for packaging applications [29]. On the other hand, that work evidenced that all PLA/PAF blends were immiscible. The PAF phase formed spheroidal domains, with average size increasing with PAF concentration and poor interfacial adhesion with surrounding PLA, especially for long-alkyl-chain PAFs. Therefore, that work evidenced that very interesting properties can be obtained if PLA is blended with a small fraction of long-alkyl-chain PAFs, and these properties may be further improved by increasing the interfacial interaction between PLA and the dispersed PAF domains.

The interfacial interaction between polymer phases is generally poor in immiscible polymer blends, and this issue can be addressed by adding a compatibilizer, which is affine or miscible in both polymer phases. The compatibilizer decreases the domain size of the dispersed phase, hindering its coalescence during mixing. The interfacial adhesion of the polymeric constituents is therefore enhanced and the final mechanical properties are consequently improved [30]. Typically, such a compatibilizer is a third polymer phase. An interesting alternative strategy to promote blend compatibility, first theorized by Ginzburg in 2005 [31], is to add solid nanoparticles. Nanoparticles can act as compatibilizers by adsorbing polymer molecules [30] and can slow down the phase separation between the blend components both when they locate at the interface and when they are preferentially segregated in one of the two polymer phases [31]. During the past few decades, many nanofillers have been considered as blend compatibilizers, such as carbon nanotubes, silica, and nanoclays [32,33].

Among the nanofillers that could be exploited as compatibilizing agents, particularly interesting are carbon-based nanofillers such as graphene, graphene oxide (GO), and reduced graphene oxide (rGO). The amphiphilic nature of GO and rGO has been exploited for the compatibilization immiscible blends such as polyamide (PA)/poly(phenylene oxide) (PPO), poly(methyl methacrylate) (PMMA)/polystyrene (PS), and poly(vinylidene fluoride) (PVDF)/thermoplastic polyurethane (TPU) blends [11]. rGO, in particular, has been an object of increasing interest as a multifunctional nanofiller enhancing not only the blend compatibilization, but also the mechanical properties and electrical conductivity of the resulting materials [11,34,35]. Moreover, GO and rGO have been proven effective in decreasing the gas permeability of polymer films, which is a key property for packaging applications [36].

Therefore, the aim of this work is twofold. The first goal is to prepare and characterize thin (50 µm) films by blending PLA with 10 wt% of poly(dodecylene furanoate) (PDoF), which is the PAF with the longest currently available alkyl chain. To the best of the authors’ knowledge, the properties of PLA/PDoF blends have never been reported in the open scientific literature. The second goal is to introduce rGO in these blends, to assess its efficacy as a blend compatibilizer and evaluate its effect on the mechanical properties, electrical conductivity, and gas barrier properties of the prepared films.

## 2. Results and Discussion

### 2.1. Characterization of rGO Nanofiller

**Figure 1:**
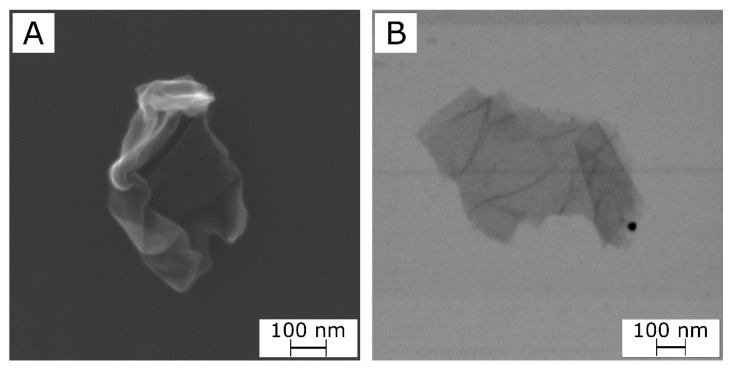
SEM (**A**) and STEM (**B**) micrographs of an rGO nanoplatelet.

Figure 1A,B show representative SEM and STEM micrographs of an isolated rGO structure after the sonication process. The SEM micrograph (Figure 1A) shows a particle with a wrinkled and folded morphology, irregular borders, and a lateral size of approx. 1 μm. The STEM micrograph (Figure 1B) was obtained by analyzing a nearly flat structure. The darker borders and zones suggest that this structure is constituted by partially overlapping rGO nanoparticles with a size of a few hundred nanometers.

**Figure 2:**
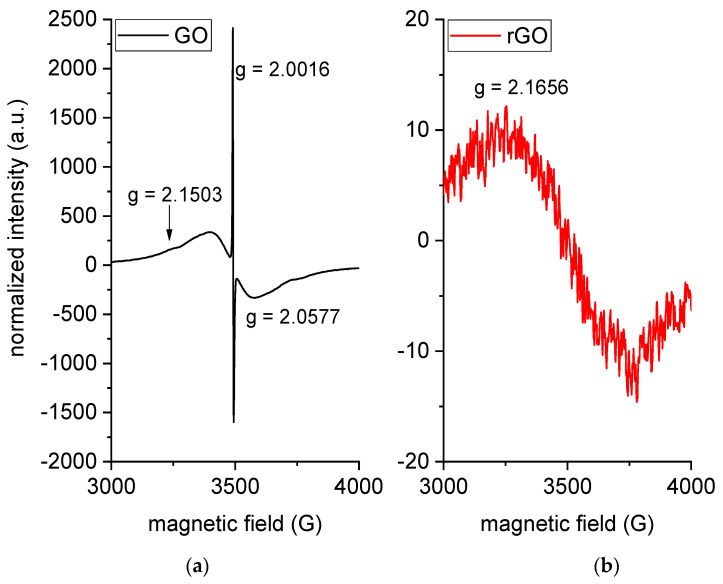
ESR spectra of GO (**a**) and rGO (**b**).

The reduction degree achieved upon treatment with HH on GO flakes was evaluated quantitatively through ESR measurements. For easier comparison, as reported in the literature, raw spectra were processed by converting the magnetic field into the corresponding Landé value (g-factor). Figure 2 shows the normalized ESR spectra of GO and rGO, where three main signals are present: a narrow and very intense peak at g = 2.0016 and a broad band (g = 2.0577) that partially overlaps to a smaller signal at g = 2.1503. The narrow peak is ascribed to oxygen-based functional groups, whereas the broad bands to unpaired spins arising from dangling bonds in the graphene structure [37]. The sharp signal associated with oxygen-containing structures is predominant in the GO structure but completely disappears in the rGO spectrum, which suggests that the GO has achieved a very high reduction degree. However, ESR probes only unpaired spins; therefore, the complete elimination of oxygen cannot be assured.

The residual signal at g = 2.1503 in GO is ascribed to chemical impurities derived by the industrial oxidation process. The signal moves to higher g values in rGO (g = 2.1656).

### 2.2. Microstructure and Spectroscopic Properties of the Prepared Films

**Figure 3:**
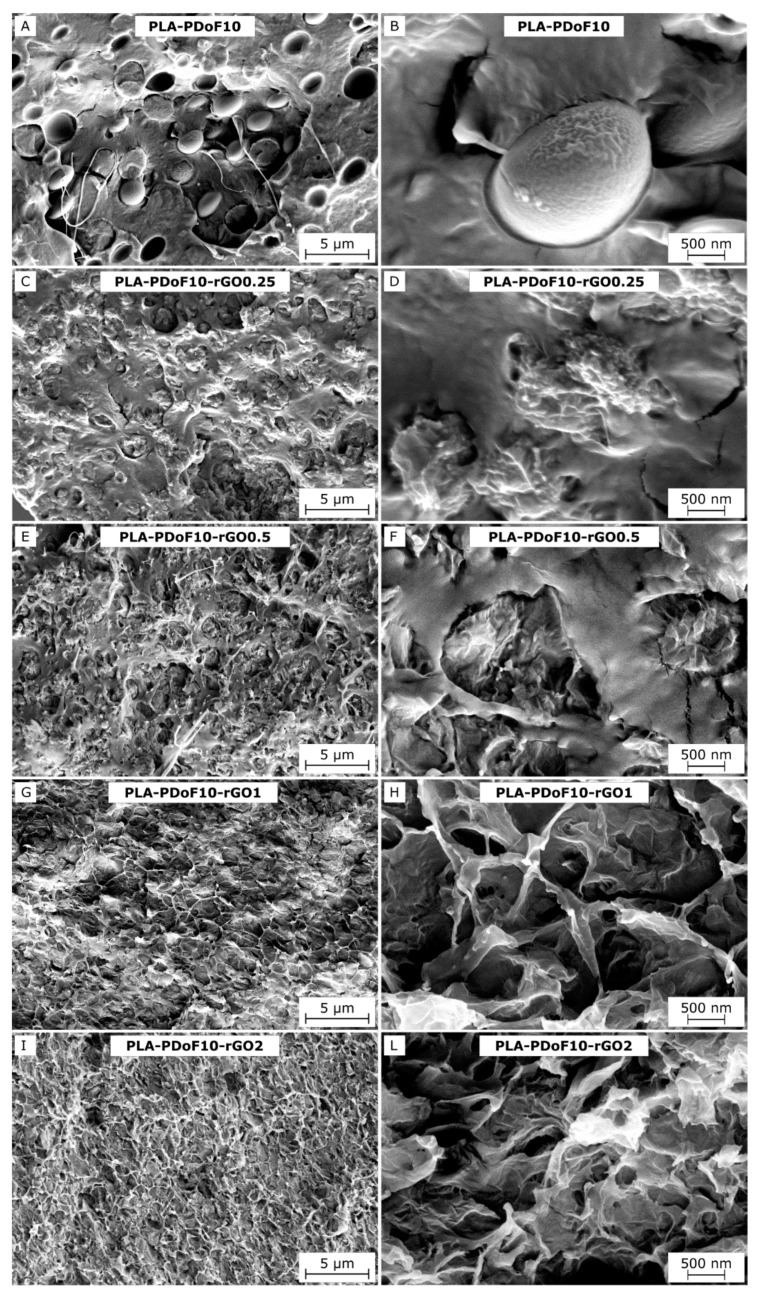
SEM micrographs of the cryofracture surface of some selected compositions at two magnification levels. (**A**,**B**) PLA-PDoF10; (**C**,**D**) PLA-PDoF10-rGO0.25; (**E**,**F**) PLA-PDoF10-rGO0.5; (**G**,**H**) PLA-PDoF10-rGO1; (**I**,**L**) PLA-PDoF10-rGO2.

Figure 3A–L show the SEM micrographs of the cryofracture surface of some selected films. From the micrographs of neat PLA-PDoF10 (Figure 3A,B), it can be noticed that PDoF and PLA are immiscible and PDoF is visible as homogeneously distributed spheroidal domains with a rather smooth surface and an average diameter of 2.6 ± 0.4 µm (measured with software ImageJ v. 1.50i). The interfacial adhesion with PLA is rather limited, as it is better observable from the micrograph at higher magnifications (Figure 3B). This is also appreciable by the fact that the cryofracture propagates mostly at the PLA–PDoF interface and more rarely across PDoF domains.

The morphology of PDoF domains changes considerably with the addition of 0.25 phr of rGO (Figure 3C,D), whereas the fracture morphology of the PLA phase does not vary appreciably. This suggests that rGO segregates preferentially in the PDoF phase rather than in PLA. With the addition of 0.25 phr of rGO, the PDoF domains become smaller (1.6 ± 0.3 µm) and rougher, while the interfacial interaction with PLA increases. The transition to a more irregular geometry and the size decrease of the PDoF domains result in a larger interfacial surface between PLA and PDoF, thus suggesting an improved compatibility between the polymer phases.

As reported in the Introduction, the use of nanofillers to enhance compatibility in polymer blends has first been theorized by Ginzburg in 2005 [31]. The rGO-induced compatibilization has been observed elsewhere in the literature, for example, in polystyrene/poly(vinyl methyl ether) blends [35] and poly(vinylidene fluoride)/polyurethane blends [11]. However, in those cases, the compatibilization resulted from the migration of rGO at the interface between the involved polymer phases, while in our case rGO appears preferentially segregated inside the PDoF phase. In any case, it has been demonstrated that nanoparticles in general can reduce the kinetics of phase separation between two polymers, even if these particles have a stronger affinity toward one of the two polymer phases [31], which is likely the case of the present work.

The morphological variation of PDoF domains due to rGO introduction is more pronounced at elevated rGO amounts, as observable from the sample with an rGO content of 0.5 phr (Figure 3E,F). Above this rGO concentration, the PDoF phase allows partial accommodation of the rGO with the surplus being distributed in the PLA matrix. This further modifies the morphology of the PDoF domains and of the fracture surface, making the detection of the PDoF domains increasingly difficult (Figure 3G,L).

**Figure 4:**
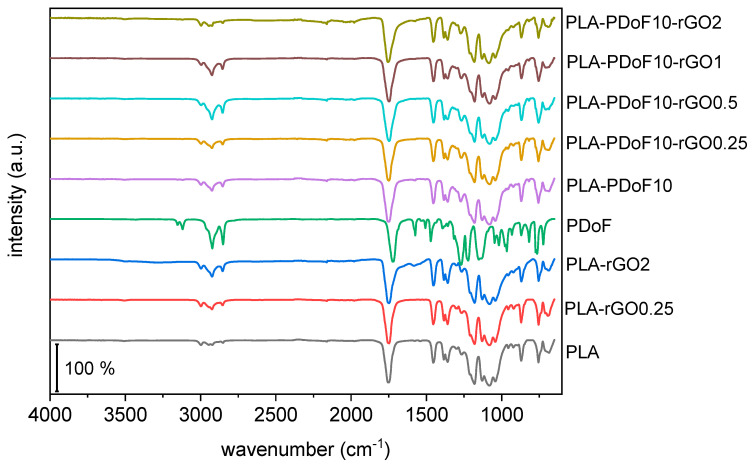
ATR-FTIR spectra of the prepared films. Spectra have been vertically translated and normalized to the most intense peak.

The microstructure of the prepared films was further studied with FTIR spectroscopy. Figure 4 presents the FTIR spectra of the prepared films after baseline correction, normalization to the most intense signal, and vertical shifting. A more detailed FTIR analysis on similar PLA-based blends with precise peak assignment has been performed in [29], while the present work reports only a general overview of the FTIR spectra.

Neat PLA, as expected, shows a small signal of C-H stretching at 2950–3000 cm^−1^ and the signals of C=O stretching at 1751 cm^−1^ and C–O–C stretching at 1180 cm^−1^ [38]. Neat PDoF shows the typical signals of poly(alkylene furanoate)s, and more specifically, the symmetrical and asymmetrical furan ring stretching at 3119 and 3152 cm^−1^, the symmetrical and asymmetrical C–H stretching of the alkyl methylene groups at 2920 and 2850 cm^−1^, the vibration of the C=C bond of furan at 1574 and 1530 cm^−1^ [39,40], ester carbonyl stretching vibration C=O at 1717 cm^−1^ [41,42], furan ring breathing at 1018 cm^−1^ and ring bending at 966 cm^−1^, approx. 820 cm^−1^ and 772 cm^−1^.

The spectra of the blends and nanocomposites show the same peaks observed in the neat PLA, due to the low weight fraction of PDoF and rGO. The presence of PDoF is observable through the variation of signals at 2920 and 2850 cm^−1^ (C–H stretching of the alkyl methylene groups) and from the right shoulder of the C=O stretch band of PLA at 1751 cm^−1^. On the other hand, no characteristic vibrations can be observed for rGO, whose FTIR spectrum generally shows few weak bands that are difficult to be appreciated in ATR mode [11]. FTIR has been sometimes used to assess the interactions of rGO with the components of a polymer blend. For example, rGO added to a poly(vinylidene fluoride)(PVDF)/thermoplastic polyurethane (TPU) blend was seen to interact with the N-H groups of TPU and the -CH_2_ dipoles of PVDF, which was evidenced via red- or blue-shifts in the corresponding signals [11]. Conversely, no red- or blue-shifts have been detected in this work by comparing the FTIR spectra of the blends and nanocomposites with those of the neat polymers, which suggests that the phases do not have a remarkable chemical interaction.

### 2.3. Thermal Properties of the Prepared Films

**Figure 5:**
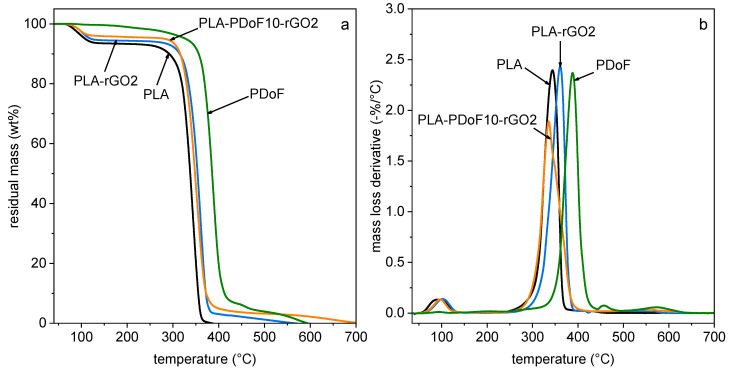
Residual mass (**a**) and mass loss derivative (**b**) as a function of temperature from TGA tests on the prepared films. Only some selected compositions are reported for clarity.

The resistance to thermal degradation of the prepared films was investigated through TGA. Representative TGA thermograms are reported in Figure 5, which shows only some selected compositions for clarity, as the trend is qualitatively similar for all curves. The most important TGA results for all samples are summarized in Table 1.

Neat PLA and all PLA-containing samples show a first mass loss between 80 °C and 120 °C, which corresponds to removal of residual solvent. Conversely, no residual solvent can be detected for neat PDoF. The total residual solvent content in the films, measured as the mass loss at 150 °C (Table 1), is approx. 4–5 wt% for all samples, even though the films have been carefully dried at room temperature for 24 h followed by 5 h at 50 °C and stored in a desiccator with silica gel. In a previous work of our group on PLA/PAF blends [29], the desiccation treatment was performed at 50 °C for only two hours, and the amount of residual solvent was approx. 6–7% for all films. The outcome of that work led to an increase in the desiccation time from 2 to 5 h in the present paper, resulting in a more efficient solvent removal. However, a certain amount of solvent still remains in the films and may affect the experimental results. The permanence of chloroform in PLA-based films was also confirmed by a recent work of this research group dealing with the high-temperature chloroform release of PLA films prepared by solvent casting [43]. In any case, all films in the present work have been prepared, desiccated, and tested in the same way and at the same time. Hence, it can be confidently assumed that the differences among the characterized compositions can be ascribed to different material properties, as already discussed in our previous work on PLA/PAF blends [29].

After solvent removal, the degradation of neat PLA shows the onset at approx. 320 °C and the maximum degradation rate at 344 °C (Table 1). The addition of 10 wt% of PDoF increases the degradation temperature of PLA of only 4 °C, even though PDoF has a remarkably higher thermal resistance than PLA. Conversely, both $T_{onset}$ and $T_{d}$ are shifted to higher temperatures with the addition of rGO, which implies that this nanofiller helps in increasing the thermal resistance of PLA, regardless of the presence of PDoF. For example, the sample PLA-PDoF10-rGO1 has a $T_{onset}$ of 331.8 °C (+11 °C compared to neat PLA) and a $T_{d}$ of 357.8 °C (+14 °C compared to neat PLA). In conclusion, TGA results evidence that PDoF does not strongly affect the thermal degradation properties of PLA, while rGO brings a small but significant positive contribution.

**Figure 6:**
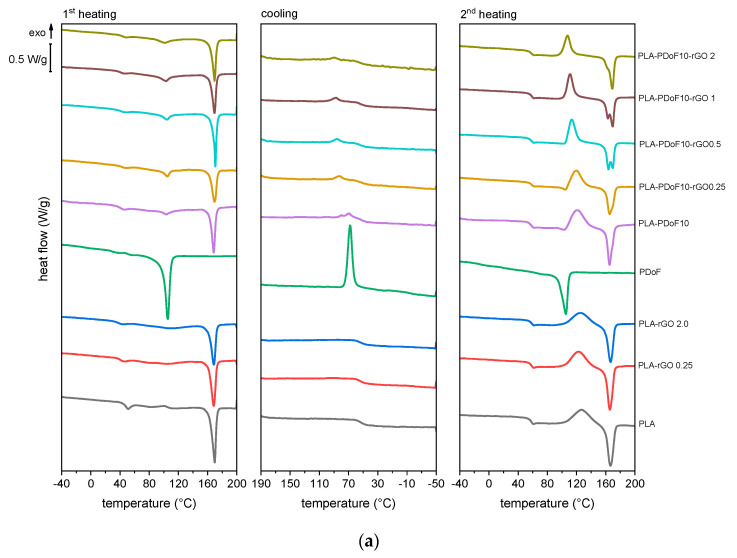

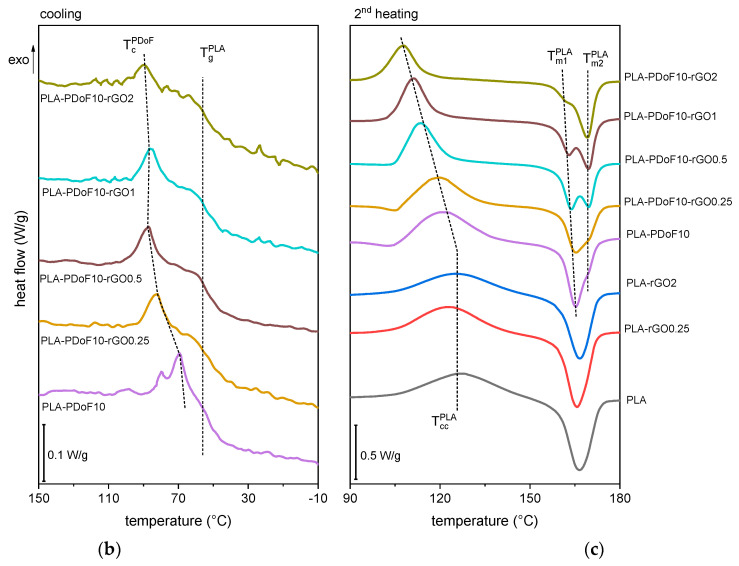
DSC thermograms of the prepared films. (**a**) Overview of the DSC thermograms in the first heating, cooling, and second heating scans; (**b**) Detail of the cooling scan with an indication of the crystallization temperature of the PDoF phase and the glass transition temperature of PLA; (**c**) Detail of the second heating scan with indication of the cold crystallization temperature of the PLA phase and the double melting behavior of PLA.

DSC thermograms of the prepared films are shown in Figure 6, while the most important DSC results are reported in Table 2. In the first heating scan, neat PLA shows glass transition at 40.9 °C, while the $T_{g}$ increases in the second heating scan (57.4 °C) due to the removal of solvent (chloroform and/or HFIP) that acts as a plasticizer, as discussed for TGA results.

For neat PLA, the glass transition is not the only relaxation detected with DSC (Figure 6a). In the first heating scan (Figure 6a), a melting peak can be observed at 169.4 °C, while the second heating scan also shows an exothermic peak at 126.1 °C, suggesting a cold crystallization event. The crystallinity degree of neat PLA in the first heating scan is 41.3%, considerably higher than that measured in the second heating scan (2.3%). This implies that the thermal treatment performed after casting to favor solvent evaporation (5 h at 50 °C) also promotes PLA crystallization, as expected, while the fast cooling scan of 10 °C/min in DSC suppresses the PLA tendency to crystallize.

The addition of rGO to PLA does not substantially modify its transition temperatures. For the samples PLA-rGO0.25 and PLA-rGO2, the $T_{g}$ is slightly lower than that of neat PLA in the first heating scan, while the values are nearly the same in the second heating scan. Moreover, the effect of rGO on the melting and cold crystallization temperatures of PLA is relatively modest. On the other hand, the addition of a small amount of rGO strongly promotes PLA crystallization, as in the second heating scan, the crystallinity degree of PLA increases from 2.3% up to 5.2% with an rGO amount of 0.25%. Almost no further increases are observable for PLA-rGO2 (${Χ}_{c}^{PLA}$ = 5.4%), as observed elsewhere in the literature [36] and ascribed to the fact that, above a certain loading threshold, the nucleating effect competes with the restriction of polymer chain mobility.

Neat PDoF shows a melting peak at 105.0 °C in both heating scans and, unlike PLA, a crystallization peak at 68.5 °C, which highlights the faster crystallization kinetics of this polymer compared to PLA. On the other hand, the $T_{g}$ is not detectable, probably due to the sensitivity limits of the instrument. The $T_{g}$ of PDoF, which should be located at approx. −5 °C [21], is not even visible by plotting the derivative of heat flow (not reported here), a mathematical expedient used to detect differences among small or very close inflection points [19]. However, the $T_{g}$ of PDoF has been detected with DMTA, as described later on in this section.

The PLA-PDoF10 sample shows the transition of both polymer phases. The $T_{g}$ of PLA phase in this sample is not remarkably different from that of neat PLA, which accounts for the immiscibility of the prepared blend, in good agreement with SEM and FTIR results. The addition of rGO into PLA/PDoF blends affects especially the cooling and the second heating scans. In the cooling scan, the crystallization temperature of PDoF is shifted to higher temperatures, as it goes from 69.3 °C of PLA-PDoF10 up to 89.7 °C of PLA-PDoF10-rGO2 (Figure 6b). This highlights the positive contribution of rGO on promoting the crystallization of PDoF and confirms the finding that rGO is preferentially located in PDoF domains, as observable by SEM.

The crystallinity degree of PLA measured in the first heating scan is quite high and comparable among the prepared samples (35–40%), while the crystallinity degree of the second heating scan is considerably lower and quite different among the samples, being 2.3% for neat PLA, 1.3% for PLA-PDoF10 and 6.3% for PLA-PDoF10-rGO2. This suggests that the thermal treatment likely promotes PLA crystallization and uniforms the crystallinity degree across the compositions, thus hiding the role played by PDoF and rGO on the final value of ${Χ}_{c}$, whereas this role is evident in the second heating scan.

In the second heating scan, the addition of rGO promotes the crystallization of PLA, as discussed before, while the addition of PDoF seems to hinder it. More interestingly, the simultaneous addition of PDoF and rGO has a more significant impact on PLA crystallinity, which generally increases with rGO content and shows a maximum of 8.5%. Additionally, in the samples containing both rGO and PDoF, an increase in rGO amount not only shifts the cold crystallization peak to lower temperatures, which is an additional sign of the increased crystallization kinetics in the solid state, but also splits the subsequent melting peak of PLA into two (Figure 6c). This double melting behavior, observed elsewhere in the literature for PLA-based nanocomposites, has been attributed to the melting of two population of crystallites: the peak at high temperature results from the melting of major lamellae formed during primary crystallization from the melt, while that at lower temperature originates from the melting of smaller lamellae formed during cold crystallization [44]. This hypothesis is also supported by the fact that both the low-temperature peak ($T_{m1}$ in Figure 6c) and the cold crystallization peak are both less intense and shifted to lower temperatures upon rGO addition, which suggests that the two peaks are relative to the same population of lamellae. Conversely, the intensity of the high-temperature peak ($T_{m2}$ in Figure 6c) increases upon rGO addition, which suggests that rGO promotes the crystallization of PLA in the cooling scan. Interestingly, the double melting behavior of PLA is clearly observable only when rGO and PDoF are present together, which suggests that this behavior may be accentuated by the interfacial interaction between PLA and the rGO-filled PDoF domains, and this is supported by the fact that the interfacial area increases upon rGO addition due to the decrease in PDoF domain size.

Of course, all films prepared in this work show high crystallinity and exhibit the thermal properties measured in the first DSC heating scan, while the differences in crystallinity and the other thermal effects detected in the second heating scan are surely not reflected on the mechanical, optical, and gas barrier properties reported hereafter. Nevertheless, the synergism between PDoF and rGO in enhancing PLA crystallinity is a remarkable phenomenon and the effects could be interesting, especially in view of the industrial scale-up of the process, which would involve the processing in the molten state. This is especially important for packaging applications, as an increase in ${Χ}_{c}$ in PLA generally leads to an improvement in stiffness, strength, heat deflection temperature (HDT), chemical resistance, and gas barrier properties [6,8,45,46].

**Figure 7:**
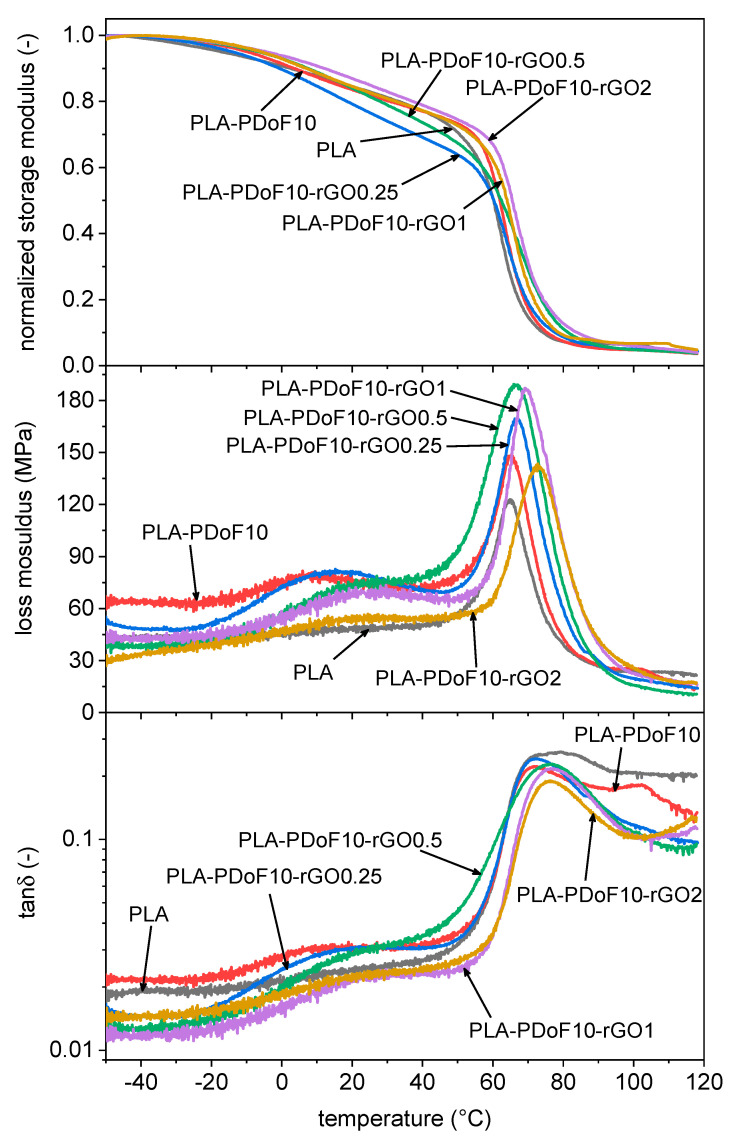
DMTA thermograms of the samples PLA, PLA-PDoF10 and PLA-PDoF10-rGOx (x = 0.25 ÷ 2 phr). Normalized storage modulus ($E^{\prime }$), loss modulus ($E^{\prime \prime }$) and tanδ as a function of temperature.

Since the DSC analysis could not evidence the glass transition of PDoF, a deeper investigation was performed with DMTA. The results of this characterization, performed with a focus on the samples containing PDoF, are shown in Figure 7. Data of storage modulus have been normalized to the value at the beginning of the test (at −50 °C), and the absolute data of $E^{\prime }$ at different temperatures are reported in Table 3. From these data, it is evident that the introduction of PDoF promotes a decrease in the values of $E^{\prime }$ compared to neat PLA in the whole investigated temperature range, while the addition of rGO compensates this effect, which is evident especially at high rGO concentrations.

For the trend of $E^{\prime }$ in the investigated temperature range, neat PLA shows a marked decrease in $E^{\prime }$ in correspondence of the glass transition, evidenced by peaks in the trends of $E^{\prime \prime }$ and tanδ. All the other compositions show very similar behavior, which is not surprising as PLA is the main component in all the prepared films. However, the compositions containing PDoF show additional signals at the glass transition and the melting of this polymer, and the effect of rGO on these transitions is arguably the most interesting result of DMTA tests. More specifically, the sample PLA-PDoF shows the glass transition of PDoF as broad peaks in the trends of $E^{\prime \prime }$ and tanδ, at approx. 5 °C. The addition of rGO shifts both these peaks to the higher temperature and decreases their intensity, which evidences the chain immobilization effect of rGO on this polymer. The shift of the $E^{\prime \prime }$ peak is also observable for PLA, especially at higher rGO loadings (1–2 phr), while the sole PDoF addition does not induce any shifts, which confirms once again the blend incompatibility.

Moreover, the tanδ signal of PLA-PDoF10 also evidences the melting transition of PDoF, occurring at approx. 102 °C, in good agreement with the DSC results. Interestingly, this peak is no longer visible after rGO addition, which could again be due to the immobilization of PDoF chains performed by rGO.

### 2.4. Mechanical Properties of the Prepared Films

**Figure 8:**
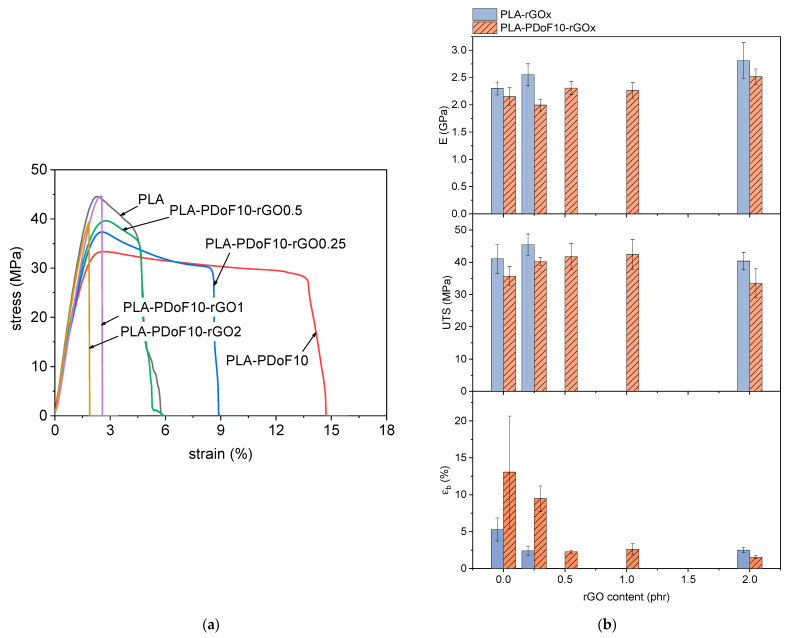
Results of the quasi-static tensile tests on the prepared films. (**a**) Representative stress–strain curves of some selected compositions; (**b**) tensile modulus (E), ultimate tensile stress (UTS) and strain at break ($\varepsilon_{b}$) as a function of the rGO content for the samples PLA-rGOx and PLA-PDoF10-rGOx (x = 0.25 ÷ 2 phr).

The main results of the tensile tests are presented in Figure 8a,b. Figure 8a shows representative stress–strain curves of some selected compositions, namely PLA and PLA-PDoF10-rGOx. The compositions PLA-rGO0.25 and PLA-rGO2 were not reported as they have a mechanical behavior qualitatively similar to that of PLA-PDoF10-rGO0.25 and PLA-PDoF10-rGO2, respectively. Additionally, Figure 8b reports the values (mean value ± standard deviation) of the elastic modulus (E), ultimate tensile stress (UTS) and strain at break ($\varepsilon_{b}$) as a function of the rGO content. The UTS, calculated as the maximum stress, was chosen as a measurement of the tensile strength of the films because of the non-uniform mechanical behavior across the compositions. In fact, as shown by Figure 8a, some compositions manifest a clear yield point, after which the stress decreases until the break, while other compositions, typically those with the highest rGO content, fail before yielding. Therefore, it is difficult to compare the mechanical strengths of the samples by considering either the stress at yield or the stress at break, whereas it is considerably more meaningful to evaluate the maximum sustainable stress, i.e., the UTS.

Neat PLA shows an elastic modulus of 2.3 GPa, a UTS of 41 MPa and an $\varepsilon_{b}$ of 5.3%. The behavior of PLA is more brittle than that reported in our previous work [29], which can be explained by the fact that the samples here have been desiccated longer to promote a more extensive solvent removal. This procedure successfully decreased the residual solvent, as demonstrated by TGA, but it also increased the PLA crystallinity, as evidenced by DSC, which explains the increase in stiffness and strength and the decrease in ductility.

Considering PLA-rGOx samples (Figure 8b), the addition of rGO to PLA increases the elastic modulus and decreases the strain at break, as commonly reported in the literature for different polymer matrices containing graphene-based nanofillers [47]. On the other hand, the UTS increases with an rGO content of 0.25 phr, and then it decreases for an rGO content of 2 phr, as the excessive nanofiller agglomeration and the consequent embrittlement cause the material to fail before yielding, as also reported in the literature for similar systems [36].

Considering now the effects of PDoF in the PLA matrix, the addition of 10 wt% of PDoF leads to a slight decrease in the elastic modulus and UTS and to a noticeable increase in the strain at break, which rises from 5.3% of neat PLA to 13.0% of PLA-PDoF10 (+145%). Although the standard deviation is quite high for the strain at break results, these findings are in good agreement with our previous work on PLA/PAF blends, which also showed a strong increase in ductility with the addition of a small fraction of long-alkyl-chain PAFs [29]. The addition of 0.25 phr to this PLA/PDoF blend increases the UTS from 35.6 MPa to 40.2 MPa (+13%) and does not significantly modify the strain at break. The increase in both the mechanical strength and the strain at break is generally indicated as a sign of blend compatibilization [11,48]. Although UTS increases upon rGO addition, it is very difficult to appreciate any variation in $\varepsilon_{b}$, mainly due to the noticeable data dispersity of PLA-PDoF10, and therefore, it is difficult to draw any conclusions from this parameter. Further additions of rGO to the PLA-PDoF10 blend promote a further increase in UTS, which is maximum for PLA-PDoF10-rGO1 (42.4 MPa). On the other hand, the UTS decreases to 33.5 MPa with an rGO content of 2 phr, which could be again due to excessive agglomeration of rGO, similarly to what was reported for PLA-rGOx samples.

Overall, the mechanical results indicate that the addition of PDoF at 10 wt% positively contributes to the ductility of PLA without significantly compromising the stiffness and strength. This increase in ductility may be more remarkable with a lower PDoF content (e.g., 5 wt%), as suggested by our previous work on other PLA/PAF blends [29], and this will be the object of upcoming studies. Moreover, the beneficial contribution of rGO on the mechanical properties of PLA and PLA-PDoF10 is rather modest. The most promising composition is PLA-PDoF-rGO0.25, although a further improvement in the mechanical properties may be obtained with a lower rGO content (e.g., 0.1 phr), to enhance the system’s homogeneity without leading to an excessive embrittlement.

### 2.5. Functional Properties of the Prepared Films

#### 2.5.1. Gas Barrier Properties

**Figure 9:**
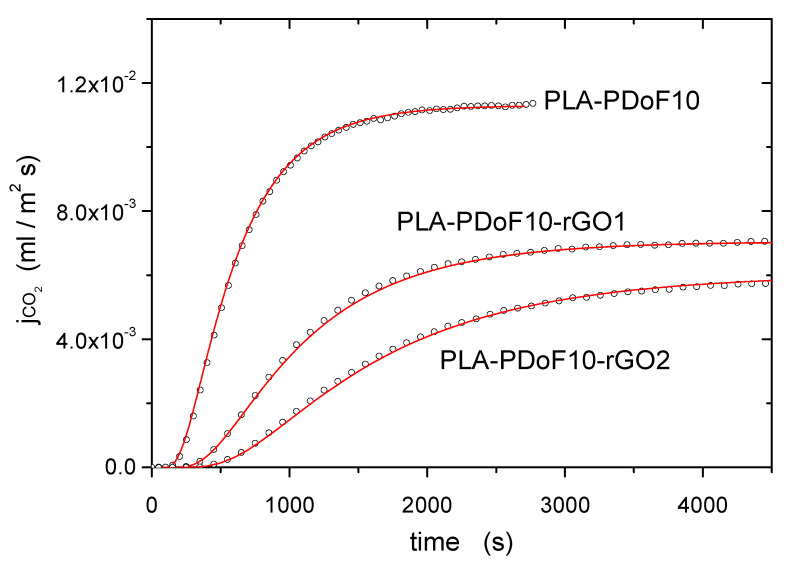
Representative trends of gas permeation flux $j_{p}\left(t\right)$ for CO_2_ obtained on the samples PLA-PDoF10, PLA-PDoF10-rGO1 and PLA-PDoF10-rGO2 (T= 298 ± 1 K, $p_{feed}=$ (45 ± 1) × 10^3^ Pa). Experimental data: open symbols. Fitting equation: solid line.

Figure 9 reports the permeation flux $j_{p}\left(t\right)$ as a function of time t obtained at T= 298 ± 1 K exposing the PLA-PDoF10, PLA-PDoF10-rGO1 and PLA-PDoF10-rGO2 film samples to CO_2_ at $p_{feed}=$ (45 ± 1) × 10^3^ Pa. Experimental data in the $j_{p}\left(t\right)$ permeation curves are reported as open symbols: experimental indetermination is inside the size of the symbols. For all tested samples, the $j_{p}\left(t\right)$ curves show an initial transient where the permeation flux value increases with time, followed by stationary transport conditions where $j_{p}\left(t\right)$ exhibits a constant ${\hat{J}}_{p}$ value. The analysis of these curves allows the evaluation of the gas permeability Φ and diffusivity D. The gas permeability Φ can be, in fact, determined by measuring the permeation flux in stationary transport conditions by the relation, reported in Equation (1) as

(1) $${\hat{J}}_{p}=\frac{1}{l}\Phi \;p_{feed}\;,$$

where l is the membrane thickness [49]. The previous approximation holds because, given the dynamic pumping conditions in the permeation chamber, it results that $p_{feed}\gg \;p_{lps}\left(t\right)$. The gas diffusivity D can then be evaluated fitting the $j_{p}\left(t\right)$ curve with the function reported in Equation (2) as

(2) $$j_{p}\left(t\right)={\hat{J}}_{p}\;\left[1+2\overset{\infty }{\underset{n=1}{\sum }}{\left(-1\right)}^{n}\;e^{-D\;n^{2}\;\pi^{2}\;t/l^{2}}\right]\;,$$

which holds because the polymer film has planar geometry and its thickness *l* is much smaller than its lateral size [49]. Lines fitting the experimental data in Figure 9 were obtained by this procedure.

**Figure 10:**
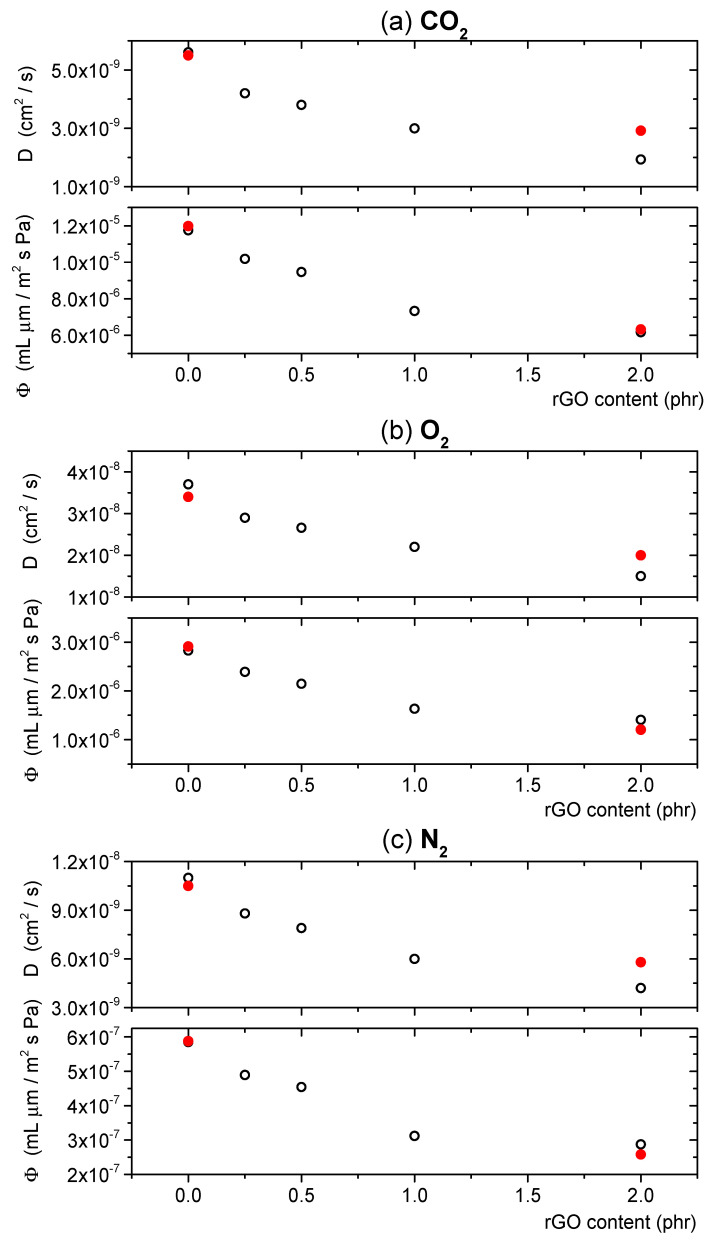
Diffusivity (D) and permeability (Φ) values pertinent to CO_2_ (**a**), O_2_ (**b**) and N_2_ (**c**) as a function of the rGO content for the samples PLA-rGOx (solid symbols) and PLA-PDoF-rGOx (open symbols) (x = 0.25 ÷ 2 phr). Experimental indeterminations are inside the size of the symbols.

The obtained diffusivity (D) and permeability (Φ) values for the three investigated gases, i.e., CO_2_, O_2,_ and N_2_, are reported in Figure 10a–c for both the PLA-rGOx samples (solid symbols) and PLA-PDoF10-rGOx samples (open symbols). Experimental indeterminations are inside the size of the symbols. For all the investigated gases, the PLA-PDoF10 sample has a slightly lower diffusivity but similar permeability value as the neat PLA film. The same behavior is also observed by comparing the gas transport properties of PLA-rGO2 and PLA-PDoF10-rGO2 nanocomposites. Although it is difficult to draw a conclusion from a test on a single composition, it seems that PDoF could contribute to enhance the gas barrier properties of PLA, but tests with greater PDoF fractions (e.g., 30 wt%) are needed to clarify the role of the PDoF phase in the gas barrier properties of the film.

Considerably more evident is the effect of rGO. The value of permeability Φ decreases by increasing the rGO content, and this decrease is always accompanied by a comparable decrease in the gas diffusivity D. This evidence clearly indicates that the improvement of the gas barrier properties is due to reduced penetrant mobility rather than to a decreased gas solubility. Additionally, the permeability reduction is of the same order of magnitude for all penetrants: given the impermeable character of the nanoplatelets, the improvement of the gas barrier properties can be attributed to longer diffusion paths for the migrating molecules in the nanocomposites.

The increase in the diffusion path length with the filler content can be explained with the Nielsen model [50]. According to this model, when platelet-like filler particles are dispersed in the polymer matrix, the effective migration path for permeating gas molecules is longer than the film thickness by a factor *τ*, called tortuosity factor. This factor is maximized when these particles form a regular stacking and their surface is parallel to the membrane surfaces; this is given in Equation (3) as

(3) $$\tau =1+\frac{1}{2}\;\alpha \;\varphi$$

where α is the aspect ratio of the filler particles and φ the filler volume fraction in the nanocomposite. The increase in the penetrant migration path decreases the effective penetrant diffusivity, as described by Equation (4) as

(4) $$D=\frac{D_{0}}{\tau }$$

where $D_{0}$ and D are the penetrant diffusivity values without and with the nanofiller, respectively.

The lateral size L of the present rGO nanoparticles is ~500 nm, as discussed for SEM results (Figure 1). Assuming a thickness W ~ 1 nm [51], then α=L/W~ 500. Assuming that the mass density of the filler particles is approximately double than that of the polymer matrix [52], then the filler volume fraction φ is approx. half of the mass fraction.

**Figure 11:**
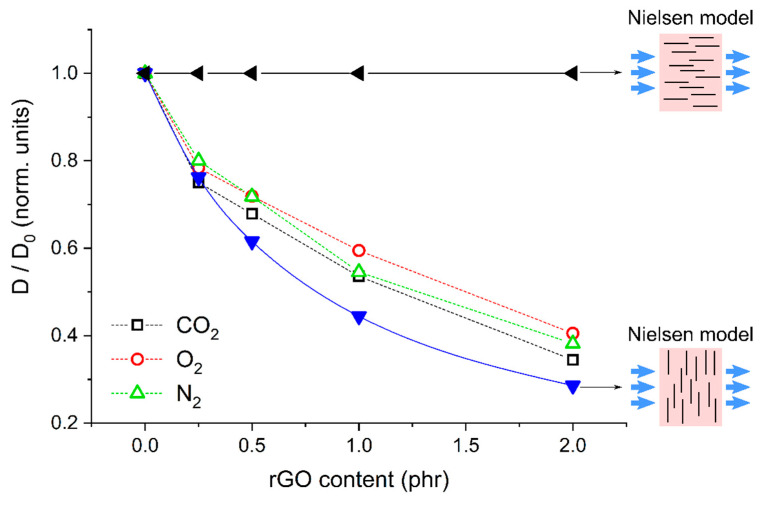
Comparison between the experimental $D/D_{0}$ values (open symbols) and those calculated through the Nielsen model in two opposite cases (solid symbols) for the samples PLA-PDoF10-rGOx (x = 0.25 ÷ 2 phr).

Figure 11 shows the values of $D/D_{0}$ obtained from the experimental diffusivity data (open symbols) on the samples PLA-PDoF10-rGOx and the values $D/D_{0}$ calculated with the Nielsen model (solid symbols) in two cases, i.e., when the rGO platelets are oriented with their surface parallel or perpendicular to the film surface. For all penetrants, the optimal $D/D_{0}$ value is slightly lower than experimental data. This can be due to several reasons, for example the fact that a fraction of the dispersed rGO is not aligned with the surface parallel to the film surface or their partial aggregation. To further increase the gas barrier performance, one should increase the filler aspect ratio [53], which may be achieved by trying to preserve the original lateral size of rGO platelets by adopting milder reduction and sonication procedures. In fact, the adopted sonication procedure was quite aggressive, but it was necessary to obtain a stable and well dispersed rGO suspension since rGO was considerably agglomerated after the reduction treatment. This problem could be avoided by studying alternative rGO preparation procedures or techniques to avoid rGO agglomeration during reduction.

#### 2.5.2. Optical Properties

**Figure 12:**
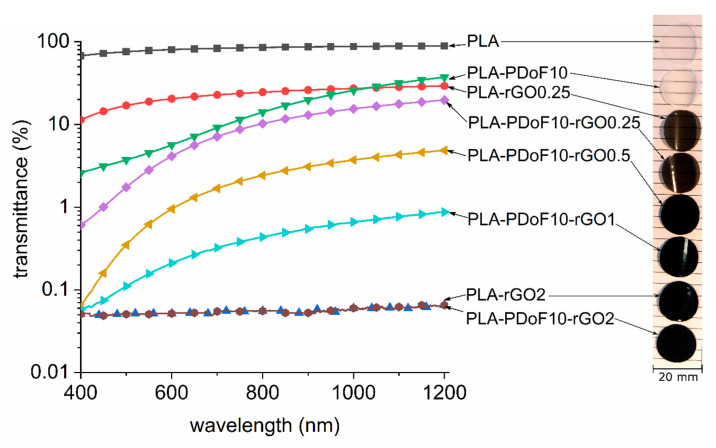
Log-scale transmittance spectra and representative pictures of the prepared films.

Complementary to other functional properties, optical transmittance measurements were acquired to investigate the effect of rGO addition to PLA-PDoF blends. Transmittance spectra of PLA, PLA-PDoF, PLA-PDoF-rGOx and PLA-rGOx are reported in Figure 12. Neat PLA film shows an almost constant transmittance, with an average value of 78 ± 4% in the visible range (400–700 nm). Upon addition of 10 wt% PDoF (sample PLA-PDoF-10), the transmittance significantly reduces to an average of 5 ± 2% in the visible range. The reduction is less remarkable in the case of rGO addition, with a reduction down to an average of 19 ± 3% in the visible range. Detrimental results are obtained upon the addition of both PDoF and rGO to PLA matrix (samples PLA-PDoF-rGOx), with average transmittances below 3% in the visible range. All these differences are qualitatively appreciable in Figure 12 by direct comparison of the samples. Considering food packaging application, the addition of graphene-containing fillers results in detrimental loss of transmittance that can significantly affect the consumer perception. However, a good compromise between technical performance and visual perception is represented by PLA-PDoF-rGO0.25 composition.

#### 2.5.3. Electrical Properties

**Figure 13:**
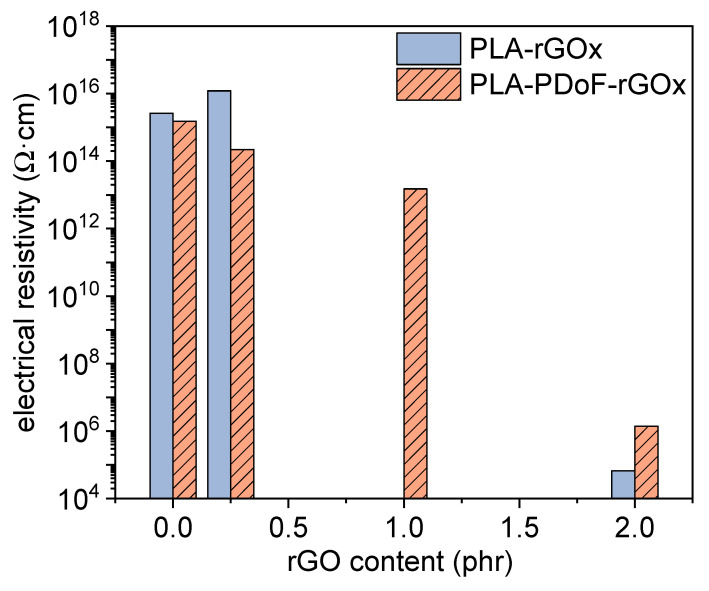
Log-scale volume electrical resistivity of the prepared films. The data dispersion was never higher than 10 Ω·cm.

Figure 13 shows the values of electrical resistivity of the samples PLA-rGOx and PLA-PDoF10-rGOx as a function of the rGO content. The measurements were repeated three times per sample and the data dispersion was never higher than 10 Ω·cm. PLA and PLA-PDoF10 do not show a significant decrease in electrical resistivity when the rGO concentration is 0.25 phr. On the other hand, a significant decrease in the electrical resistivity is observed for the samples with 2 phr of rGO. In fact, the resistivity of neat PLA decreases from 2.9 × 10^15^ Ω·cm to 6.7 × 10^4^ Ω·cm, while that of PLA-PDoF10 decreases from 1.7 × 10^16^ to 1.4 × 10^6^ Ω·cm after adding 2 phr of rGO. Therefore, the percolation threshold is in both cases between 1 and 2 phr.

The identified percolation threshold is higher than that reported in the literature for graphene-based nanocomposites, which is usually in the range 0.1–0.5 wt% [34,47,54,55,56]. The reason behind a high percolation threshold could be found in three main causes, namely the low electrical conductivity of the prepared rGO, the small size and/or wrinkled morphology of rGO sheets, and the poor dispersion of the nanofiller, which generally lowers the filler aspect ratio (area/thickness). Since the ESR tests demonstrated the high degree of reduction of the prepared rGO, the nanofiller used in this work likely has a high electrical conductivity. On the other hand, SEM and STEM micrographs confirmed the small lateral dimension of rGO and the wrinkled morphology, which was observed in some micrographs. Moreover, in the samples PLA-PDoF10-rGOx, the rGO is preferentially distributed in the dispersed PDoF phase rather than in the PLA matrix, which further limits a uniform dispersion of the nanofiller. This also explains the higher electrical resistivity of PLA-PDoF10-rGO2 compared to PLA-rGO2.

In any case, rGO does modify the electrical behavior of the prepared films. Films for packaging and electronics are often classified according to the Standard ANSI/EIA-541, “Packaging Materials Standards for electrostatic discharge (ESD) sensitive Items”. The standard classifies the materials as insulative (*ρ* higher than 10^11^ Ω·cm), dissipative (*ρ* between 10^4^ Ω·cm and 10^11^ Ω·cm) and conductive (*ρ* lower than 10^4^ Ω·cm) and defines them as antistatic materials that are either dissipative or conductive. According to this classification, the films prepared in this work are insulative with an rGO loading lower than 1 phr and dissipative (and antistatic) with an rGO loading equal to 2 phr.

## 3. Materials and Methods

### 3.1. Materials

The poly(lactic acid) (PLA) grade used in this work is Ingeo™ Biopolymer 4032D, provided by NatureWorks LLC (Minnetonka, MN, USA) in the form of granules. According to the producer’s technical datasheet, it is characterized by a D-lactic acid content of 2%, a specific gravity of 1.24 g/cm^3^, a melt flow index (MFI) of 7 g/10 min (210 °C, 2.16 kg), and a melting point of 155–170 °C. Poly(1,12-dodecylene 2,5-furandicarboxylate) (PDoF) was synthesized via a two-step polycondensation from 2,5-dimethylfuran-dicarboxylate and 1,12-dodecamethylene glycol, and as reported in the work of Papageorgiou et al. [21]. It shows a glass transition temperature ($T_{g}$) of −5 °C and a melting temperature ($T_{m}$) of 111 °C.

A water suspension of graphene oxide (GO) provided by Graphenea (San Sebastián, Spain) (concentration 4 mg/mL, pH value 2.2–2.5, GO monolayer content >95%, particle size <10 µm) was used to synthesize rGO. Hydrazine hydrate (HH) reagent grade (CAS Number 10217-52-4) was purchased from Sigma Aldrich and used as received. Chloroform (HPLC grade, CAS 67-66-3) and hexafluoroisopropanol (HFIP) (RPE grade, CAS 920-66-1) were purchased from Carlo Erba Reagents S.r.l. (Milano, Italy) and used as received.

### 3.2. Sample Preparation

#### 3.2.1. Synthesis of rGO

The reduction of GO to obtain rGO was carried out with a procedure similar to that reported in [11]. Twenty milliliters of GO solution were added to a round bottom flask containing 180 mL deionized water (DI). Then, HH was added to reach a HH:GO mass ratio of 1:1. The suspension was stirred under reflux conditions at 100 °C for 24 h, and after this time, the reduction reaction took place, as demonstrated by the evident agglomeration of the filler. The suspension was left cooling to room temperature and filtered with filter paper. The filtrate was washed thoroughly with DI and dried overnight in a ventilated oven at 50 °C.

#### 3.2.2. Preparation of Nanocomposite Films

PLA/PDoF/rGO nanocomposite films were prepared via solvent casting to avoid any possible transesterification reaction of PDoF at high temperature [19]. PLA and PDoF were dried at 50 °C overnight and dissolved in a mixture of chloroform and HFIP (9:1 vol:vol), since this solvent mixture has been reported to dissolve both PLA and furan-based polyesters [37]. The polymer concentration in the solution was 1 g of polymer in 25 mL of solvent, as this concentration was proven suitable for film casting [37]. The obtained solutions were magnetically stirred at 300 rpm at 50 °C for 2 h and, after this time, a certain amount of rGO suspension was poured slowly into the polymer solution to reach the desired rGO concentration. To prepare the rGO suspension, a proper amount of rGO was redispersed in chloroform (1 mg/mL) and sonicated for 3 h with an ultrasonic tip (UP-400S, Hielscher Ultrasonics GmbH, Teltow, Germany), just before being poured into the polymer solution. Such an aggressive sonication procedure was necessary to reach a stable and well dispersed rGO suspension, as rGO resulted considerably agglomerated after reduction. A variable volume of rGO suspension was added to the polymer solution to prepare nanocomposites with variable final rGO concentration. The PLA/PDoF/rGO suspensions were further magnetically stirred at 300 rpm at 50 °C for 3 h, then mildly ultrasonicated for 20 min in a Labsonic LBS1 bath (Falc Instruments Srl, Bergamo, Italy), casted in glass Petri dishes, and left 24 h at room temperature and 5 h at 50 °C, to remove the solvent. The process led to the production of free-standing films with a thickness of approx. 50 µm and a variable content of rGO. The prepared films with their nominal weight composition are listed in Table 4. Neat PLA and PLA/rGO films without PDoF were also prepared for comparison. A neat PDoF film was produced only for the thermal characterization, due to the scarcity of material available.

### 3.3. Experimental Techniques

#### 3.3.1. Microstructural and Spectroscopic Properties

The morphology of the rGO nanofiller was studied by Field Emission Scanning Electron Microscopy (FE-SEM) using a JEOL JSM-7001F microscope operating at 2 kV, in SEM and scanning-transmission electron microscopy (STEM) modes. rGO powders were sonicated in chloroform with the same sonication procedure applied to prepare the nanocomposite films (see Section 2.2), and then they were further dispersed in chloroform to obtain a nearly transparent and very diluted mixture. Drops were deposited on a Si wafer and analyzed after solvent evaporation.

Electron Spin Resonance (ESR) measurements were acquired with a Bruker EMX-6/1 (200 mW Gunn source, X-band 9.77 GHz) at room temperature on GO and rGO powders upon complete desiccation in an oven at 80 °C for 4 h. Spectra were acquired with the same parameters listed in [57], and the signal was normalized on the sample mass.

SEM micrographs of cryofracture surfaces of the prepared films were obtained with an FE-SEM Zeiss Supra 60 (Carl Zeiss AG, Oberkochen, Germany) at different magnification levels after Pt-Pd sputtering.

Fourier-transformed infrared (FTIR) spectroscopy was carried out on the prepared films in attenuated total reflectance (ATR) mode with a Perkin-Elmer Spectrum One instrument (Perkin Elmer GmbH, Waltham, MA, US). Data were collected in the wavenumber range 650–4000 cm^−1^, and 100 scans were superimposed for each spectrum (resolution 4 cm^−1^).

#### 3.3.2. Thermal Properties of the Prepared Films

Differential scanning calorimetry (DSC) was performed with a Mettler DSC 30 calorimeter (Mettler Toledo, Inc., Columbus, OH, USA) at 10 °C/min. Specimens of approx. 5 mg were subjected to a first heating scan, a cooling scan, and a second heating scan between −50 and 200 °C, with a nitrogen flow of 100 mL/min. One specimen was tested for each composition. The test allowed measurement of the glass transition temperature ($T_{g}$) and the melting, cold crystallization, and crystallization temperatures ($T_{m}$, $T_{cc}$, $T_{c}$) and enthalpy values ($\Delta H_{m}$, $\Delta H_{cc}$, $\Delta H_{c}$) of PLA and PDoF phases. Cold crystallization was intended as the crystallization occurring in the heating scan, at a temperature comprised between $T_{g}$ and $T_{m}$, while crystallization was considered as the transition occurring in the cooling scan. The crystallinity degree of PLA (${Χ}_{c}^{PLA}$) in the prepared films was calculated with data of both heating scans through Equation (5):

(5) $${Χ}_{c}^{PLA}=\frac{\Delta H_{m}^{PLA}-\Delta H_{cc}^{PLA}}{w\cdot \Delta H_{0}^{PLA}}\cdot 100$$

where w is the mass fraction of PLA and $\Delta H_{0}^{PLA}$ is the theoretical melting enthalpy of fully crystalline PLA, equal to 93.7 J/g [58].

Thermogravimetric analysis (TGA) was carried out with a Q5000IR thermobalance (TA Instruments, Inc., New Castle, DE, USA). Specimens of approx. 4 mg were tested at 10 °C/min up to 700 °C, under a nitrogen flow of 10 mL/min. TGA tests allowed determining (i) the residual mass at 150 °C ($m_{150{}^{\circ}C}$) after the complete removal of the residual solvent, (ii) the onset degradation temperature ($T_{onset}$), evaluated with the tangent method, and (iii) the peak degradation temperature ($T_{d}$), considered as the peak of the mass loss derivative (DTG) curve and corresponding to the maximum degradation rate.

Dynamic mechanical thermal analysis (DMTA) was carried out with a TA Instruments (New Castle, DE, USA) Q800 DMA analyzer in tensile mode on rectangular specimens with in-plane dimensions of 30 × 4 mm^2^, mounted on the instrument with a gauge length of 10 mm, calculated as the distance between the grips. Storage modulus ($E^{\prime }$), loss modulus ($E^{\prime \prime }$) and loss factor (tanδ) were determined between −50 °C and 120 °C, at a heating rate of 3 °C/min, a strain amplitude of 0.05%, and a frequency of 1 Hz.

#### 3.3.3. Mechanical Properties of the Prepared Films

Quasi-static tensile tests were performed at room temperature with an electromechanical universal testing machine Instron 5969 (Instron, Norwood, MA, USA), equipped with a 100 N load cell. Rectangular specimens with nominal in-plane dimensions of 80 × 5 mm^2^ were cut from the prepared films and glued onto paper frames to ease their handling. Specimens were mounted on the testing machine with a gauge length of 50 mm, measured as the distance between the grips, and tested at 10 mm/min. At least five specimens were tested per composition. These tests allowed the measurement of the elastic modulus (E), considered as the slope of the stress–strain curve in the initial linear region, of the stress and the strain at yield ($\sigma_{y}$, $\varepsilon_{y}$), and of the stress and strain at break $(\sigma_{b}$, $\varepsilon_{b}$).

#### 3.3.4. Functional Properties of the Prepared Films

The gas barrier properties of the prepared films were studied at 298 ± 1 K by the gas phase permeation technique in the dead-end configuration using specimens shaped in the form of a thin disc with a diameter of 13 mm, which have been die-cut from the casted films. Permeation tests were carried out using two test gases, i.e., high purity CO_2_ and a dry mixture of 20% O_2_ + 80% N_2_ (synthetic air). At time t= 0, the feed side of the disc sample was exposed to the test gas kept at constant pressure $p_{hps}$ (*hps*: high pressure side). Gas molecules are absorbed by the polymer layer surface, diffuse down to their concentration gradient to the opposite side of the polymer disc, and desorb in a vacuum test chamber. In this chamber, desorbed molecules form a rarefied gas with partial pressure $p_{lps}$ (*lps*: low pressure side). The test chamber is kept under continuous pumping conditions during the experimental run and the $p_{lps}\left(t\right)$ value is measured as a function of time t by a calibrated Quadrupole Mass Spectrometer (QMS). In dynamic pumping conditions, $p_{lps}\left(t\right)$ provides a measurement of the gas permeation flux $j_{p}\left(t\right)$ according to Equation (6):

(6) $$j_{p}\left(t\right)=\frac{1}{A}\;\frac{1}{R\cdot T_{C}}\;s_{p}\cdot p_{lps}\left(t\right)$$

where A is the effective membrane surface area, $s_{p}$ the pumping speed of the vacuum system, R the universal gas constant and $T_{C}$ the temperature of the vacuum test chamber which is ~298 K. Details on the experimental apparatus and test procedures are described elsewhere [49].

Electrical resistivity was measured in a four-point configuration, according to the standard ASTM D4496-04, on rectangular specimens with in-plane dimensions of 10 × 50 mm^2^ cut out the prepared films. A DC voltage generator ISO-Tech IPS 303DD (Milano, Italy) was connected to the specimen, an ammeter was connected in series to measure the flowing current, and a voltmeter was connected to the two inner electrodes to measure the voltage drop. The volume resistivity ρ (Ω·cm) was measured through Equation (7):

(7) $$\rho =R\frac{w\cdot t}{l}$$

where R is the resistance calculated as the slope of the voltage–current plot, linear in the measurement range; w and t are the specimen width and the thickness, respectively; and l is the distance between the inner electrodes, equal to 3.69 mm. This configuration allowed the measurement of resistivity values up to 10^7^ Ω·cm, while the resistivity of more insulating films was measured using a Keithley 6517A electrometer/high-resistance meter (Cleveland, OH, USA) and an 8009 resistivity test fixture at room temperature, according to ASTM D257. In this test, a constant voltage of 50 V was applied to circular samples with a diameter of approx. 70 mm.

Optical characterization was carried out with a Jasco V-570 dual-beam spectrophotometer. Transmittance measurements were acquired in the 400–1200 nm visible-near infrared (Vis-NIR) range with a 400 nm/min acquisition speed and 2 nm excitation bandwidth.

## 4. Conclusions

In this work, for the first time, PLA/PDoF/rGO nanocomposite films were prepared by solution casting and their properties were investigated as a function of the presence of PDoF (10 wt%) and the rGO content (0.25 to 2 phr) with microstructural, spectroscopic, thermal, mechanical, electrical, optical, and gas phase permeation techniques. The characterization of rGO showed that the reduction treatment and the following sonication procedure resulted in rGO nanoparticles with a very high degree of reduction and small lateral dimensions (few hundreds of nanometers). The microstructural characterization of the films showed that PLA and PDoF were immiscible and the PDoF was present as spheroidal domains with dimensions of 2.6 ± 0.4 µm. The addition of rGO, which preferentially segregated in the PDoF phase, resulted in smaller (1.6 ± 0.3 µm) and more irregular PDoF domains that also showed a higher interfacial interaction with PLA, which suggested that rGO can act as a compatibilizer for this blend.

Considering the thermal properties, PDoF did not considerably modify the thermal degradation resistance of PLA, as assessed through TGA, whereas the addition of rGO increased both the onset and the peak degradation temperatures. DSC tests showed that rGO increased PLA crystallinity and, more interestingly, this phenomenon was more marked when PDoF was also present, probably due to effects related to the increased interfacial interaction between PLA and rGO-filled PDoF domains, thus evidencing a synergism between PDoF and rGO in accelerating the crystallization kinetics of PLA. This phenomenon was evident only in the second heating scan, and therefore it did not reflect the crystallinity and the microstructure of the samples subjected to the rest of the characterization, which instead showed exceptionally high and similar degrees of crystallinity due to the thermal treatment, as highlighted in the first DSC heating scan. Nevertheless, this remarkable synergic effect of PDoF and rGO in promoting PLA crystallization may be interesting in view of an industrial scale-up of the process, which would involve the processing from the melt. The thermal transitions of PDoF were better observed through DMTA, which showed that the E″ and tanδ peaks at the glass transition of PDoF, observed at approx. 5 °C, were shifted to higher temperature upon rGO addition.

The mechanical tests evidenced that the addition of 10 wt% of PDoF to PLA increased the strain at break, which rose from 5.3% of neat PLA to 13.0% of PLA-PDoF10 (+145%). The addition of 0.25 phr to this PLA/PDoF blend increased the UTS from 35.6 MPa to 40.2 MPa (+13%) and did not significantly modify the strain at break. On the other hand, the UTS decreased to 33.5 MPa with an rGO content of 2 phr, likely due to excessive rGO agglomeration.

For the functional properties, the addition of both PDoF and rGO strongly modified the optical transparency of PLA, with an average transmittance below 3% in the visible range starting from 0.25 phr of rGO. Moreover, the electrical resistivity decreased with an increase in rGO content, and the relatively high percolation threshold (between 1 and 2 phr) was probably linked to the low aspect ratio of rGO nanosheets and their preferential distribution in PDoF domains. Finally, the investigation of gas barrier properties evidenced that the addition of 10 wt% of PDoF did not strongly modify the permeability of PLA, even though the slight decrease in diffusivity of PLA-PDoF10 compared to PLA suggested that a higher fraction of PDoF could be beneficial to the gas barrier properties. Conversely, the decrease in the diffusivity and permeability values promoted by rGO was remarkable for all the investigated gases. The application of the Nielsen model suggested that a further increase in the gas barrier properties may be achieved by increasing the orientation of rGO nanosheets parallel to the film plane and by enhancing the aspect ratio of rGO nanosheets, obtainable by optimizing the reduction and sonication treatments to try to limit agglomeration and fragmentation.

The results also evidence that an increasing fraction of rGO may promote agglomeration. In fact, rGO agglomeration at a concentration of 2 phr may explain the reduction in tensile strength for the sample PLA-PDoF10-rGO2 compared to PLA-PDoF10-rGO1, but also the lower thermal resistance in TGA and crystallinity in the second DSC heating scan. Moreover, agglomeration may also have influenced the electrical performance, where a better filler dispersion may have decreased the electrical percolation threshold, and the gas transport properties, where agglomeration may be among the causes for a non-optimal gas barrier performance. Therefore, the optimal concentration of rGO for this blend is lower than 2 phr, as it is difficult to reach a proper filler dispersion at this concentration.

Overall, the presented results highlight the positive and sometimes synergistic role of PDoF and rGO in tuning the thermomechanical and functional properties of PLA, with simultaneous enhancement of ductility, crystallization kinetics, and gas barrier properties, thus obtaining polymer films with balanced properties and promising for packaging applications. For packaging applications, rGO proved to be an interesting multifunctional nanofiller for this blend, especially for enhancing the gas barrier and the antistatic properties, but it could also be useful to enhance the crystallization kinetics of PLA, thus further increasing the mechanical and gas barrier performance. The characterization also evidenced that even better properties could be achieved by decreasing the weight fraction of both PDoF and rGO to reach a finer dispersion and lower agglomeration of the dispersed phases. These considerations may apply not only to this system, but more in general to polymer blends for packaging applications, where adding a multifunctional nanofiller may solve multiple issues. The addition of the proper nanofiller in the right concentration may be an economical and environmentally friendly strategy to improve blend compatibility, enhance mechanical properties, and provide functional features, such as a tunable optical transparency, and interesting antistatic and gas barrier performance.

## Figures and Tables

**Table 1:** Main results of the TGA tests on the prepared samples.

Sample	$m_{150{}^{\circ}C}\;(\%)$	$T_{onset}\;({}^{\circ}C)$	$T_{d}\;(\%)$
PLA	93.9	320.5	343.9
PLA-rGO0.25	95.6	329.5	362.5
PLA-rGO2	94.5	331.4	360.9
PDoF	99.6	366.5	388.7
PLA-PDoF10	95.2	319.3	347.9
PLA-PDoF10-rGO0.25	94.5	322.3	352.6
PLA-PDoF10-rGO0.5	94.3	330.2	357.5
PLA-PDoF10-rGO1	95.1	331.8	357.8
PLA-PDoF10-rGO2	95.9	324.0	352.1

$m_{150{}^{\circ}C}$ = residual mass at 150 °C; $T_{onset}$ = onset degradation temperature; $T_{d}$ = degradation temperature (peak of the mass loss derivative signal).

**Table 2:** Main results of the DSC tests on the prepared samples.

	Property/Sample	PLA	PLA-rGO0.25	PLA-rGO2	PDoF	PLA-PDoF10	PLA-PDoF10-rGO0.25	PLA-PDoF10-rGO0.5	PLA-PDoF10-rGO1	PLA-PDoF10-rGO2
**h1**	$T_{g}^{PDoF}\;$(°C)	-	-	-	-	-	-	-	-	-
	$T_{g}^{PLA}\;$(°C)	40.9	40.0	39.2	-	40.4	42.7	40.9	41.5	43.3
	$T_{m}^{PDoF}\;$(°C)	-	-	-	105.0	103.9	104.5	103.7	102.9	101.3
	$\Delta H_{m}^{PDoF}\;$(J/g)	-	-	-	88.6	6.9	8.0	9.4	10.2	11.5
	$T_{m}^{PLA}\;$(°C)	169.4	168.1	168.3	-	168.1	169.2	169.8	169.0	169.0
	$\Delta H_{m}^{PLA}\;$(J/g)	38.7	36.1	33.1	-	30.3	26.9	29.3	28.1	28.9
	${Χ}_{c}^{PLA}$ (%)	41.3	38.6	36.0	-	35.9	32.0	34.9	33.7	35.0
**c**	$T_{c}^{PDoF}\;$(°C)	-	-	-	68.5	69.3	82.0	86.3	87.7	89.7
	$\Delta H_{c}^{PDoF}\;$(J/g)	-	-	-	59.0	5.8	3.7	3.8	5.7	3.5
**h2**	$T_{g}^{PDoF}\;$(°C)	-	-	-	-	-	-	-	-	-
	$T_{g}^{PLA}\;$(°C)	57.4	57.9	57.9	-	58.1	58.0	57.8	58.4	57.8
	$T_{m}^{PDoF}\;$(°C)	-	-	-	105.0	103.1	104.4	-	-	-
	$\Delta H_{m}^{PDoF}\;$(J/g)	-	-	-	78.3	2.1	3.2	-	-	-
	$T_{cc}^{PLA}\;$(°C)	126.1	122.1	125.1	-	121.2	120.0	113.5	111.3	107.9
	$\Delta H_{cc}^{PLA}\;$(J/g)	38.0	37.7	32.7	-	34.6	26.6	28.3	26.5	25.2
	$T_{m}^{PLA}\;$(°C)	166.1	165.4	166.4	-	165.1	164.9	163.4	169.2	168.7
	$\Delta H_{m}^{PLA}\;$(J/g)	40.2	42.6	37.7	-	35.7	30.6	33.8	33.6	30.4
	${Χ}_{c}^{PLA}$ (%)	2.3	5.2	5.4	-	1.3	4.8	6.6	8.5	6.3

h1 = first heating scan; c = cooling scan; h2 = second heating scan; $T_{g}^{PDoF}$= glass transition temperature of PDoF;$\;T_{g}^{PLA}$ = glass transition temperature of PLA;$\;T_{m}^{PDoF}$ = melting temperature of PDoF; $\Delta H_{m}^{PDoF}$ = melting enthalpy of PDoF;$\;T_{m}^{PLA}$ = melting temperature of PLA (peak temperature); $\Delta H_{m}^{PLA}$ = total melting enthalpy of PLA;$\;T_{c}^{PDoF}$ = crystallization temperature of PDoF; $\Delta H_{c}^{PDoF}$ = crystallization enthalpy of PDoF; $T_{cc}^{PLA}$ = cold crystallization temperature of PLA; $\Delta H_{cc}^{PLA}$ = cold crystallization enthalpy of PLA; ${Χ}_{c}^{PLA}$ = crystallinity degree of PLA; - = not detectable.

**Table 3:** Main results of the DMTA tests on the prepared samples.

Sample	${E^{\prime }}_{-50{}^{\circ}C}\;(\mathrm{GPa})$	${E^{\prime }}_{30{}^{\circ}C}\;(\mathrm{GPa})$	${E^{\prime }}_{100{}^{\circ}C}\;(\mathrm{GPa})$	$T_{g\_E\prime \prime }^{PLA}\;({}^{\circ}C)$
PLA	3.4	2.7	0.16	64.8
PLA-PDoF10	2.9	2.4	0.14	64.9
PLA-PDoF10-rGO0.25	3.3	2.5	0.17	66.8
PLA-PDoF10-rGO0.5	3.8	3.0	0.18	66.3
PLA-PDoF10-rGO1	3.6	3.0	0.22	69.2
PLA-PDoF10-rGO2	3.6	3.1	0.24	72.2

${E^{\prime }}_{-50{}^{\circ}C}$ = value of *E*′ at −50 °C; ${E^{\prime }}_{30{}^{\circ}C}$ = value of *E*′ at 30 °C; ${E^{\prime }}_{100{}^{\circ}C}$ = value of *E*′ at 100 °C. $T_{g\_E\prime \prime }^{PLA}$ = *E*″ peak temperature at the glass transition of PLA.

**Table 4:** List of prepared samples with nominal weight composition.

Sample	PLA (wt%) *	PDoF (wt%) *	rGO (phr) **
PLA	100	0	0
PLA-rGO0.25	100	0	0.25
PLA-rGO2	100	0	2
PDoF	0	100	0
PLA-PDoF10	90	10	0
PLA-PDoF10-rGO0.25	90	10	0.25
PLA-PDoF10-rGO0.5	90	10	0.5
PLA-PDoF10-rGO1	90	10	1
PLA-PDoF10-rGO2	90	10	2

* weight fractions of PLA and PDoF sum up to 100%; ** phr = parts per hundred resin (PLA + PDoF).

## Data Availability

The data presented in this study are available on request from the corresponding author.

## Sample Availability

Samples of all compounds are available from the authors.
